# Feasibility study of early prediction of postoperative MRI findings for knee stability after anterior cruciate ligament reconstruction

**DOI:** 10.1186/s12891-021-04507-y

**Published:** 2021-07-30

**Authors:** Jianqiang Zhang, Jiyao Ma, Juan Huang, Guoliang Wang, Yilong Huang, Zhenhui Li, Jun Yan, Xiaomin Zeng, Hongli Zhu, Wei Zhao, Yanlin Li, Bo He

**Affiliations:** 1 Medical Imaging Department, First Affiliated Hospital of Kunming Medical University, Kunming Medical University, Kunming, China; 2 Medical Imaging Department, Yunnan Cancer Hospital &, The Third Affiliated Hospital of Kunming Medical University, Kunming, China

**Keywords:** Anterior cruciate ligament (ACL) reconstruction, Stability of knee joint, Postoperative magnetic resonance (MR) examination, MRI findings early

## Abstract

**Background:**

At present, the most effective and mature treatment after ACL injury and tear is ACL reconstruction, but the rehabilitation process after ACL reconstruction that is very long, so it is very important to find early MRI positive findings of knee instability.

**Methods:**

We retrospectively collected the clinical and imaging data of 70 patients who underwent ACL reconstruction from January 2016 to December 2019; Based on clinical criteria, the patients were divided into a stable group (*n* = 57) and an unstable group (*n* = 13); We measured the MRI imaging evaluation indexes, including the position of the bone tunnel, graft status, and the anatomical factors; Statistical methods were used to compare the differences of imaging evaluation indexes between the two groups; The prediction equation was constructed and ROC curve was used to compare the prediction efficiency of independent prediction factors and prediction equation.

**Results:**

There were significant differences in the abnormal position of tibial tunnel entrance, percentage of the position of tibial tunnel entrance, position of tibial tunnel exit, lateral tibial posterior slope (LTPS), width of intercondylar notch between stable knee joint group and unstable knee joint group after ACL reconstruction (*P* < 0.05); The position of tibial tunnel exits and the lateral tibial posterior slope (LTPS) and the sagittal obliquity of the graft were independent predictors among surgical factors and self-anatomical factors (*P* < 0.05); The prediction equation of postoperative knee stability was established: Logit(P) = -1.067–0.231*position of tibial tunnel exit + 0.509*lateral tibial posterior slope (LTPS)-2.105*sagittal obliquity of the graft; The prediction equation predicted that the AUC of knee instability was 0.915, the sensitivity was 84.6%, and the specificity was 91.2%.

**Conclusions:**

We found that abnormalities of the position of the exit of the bone tunnel, lateral tibial posterior slope (LTPS) and sagittal obliquity of the graft were the early MRI positive findings of knee instability after ACL reconstruction. It is helpful for clinicians to predict the stability of knee joint after ACL reconstruction.

## Introduction

Anterior cruciate ligament (ACL) and posterior cruciate ligament (PCL) are important static structures that maintain the stability of the knee joint. Their roles are to connect the femur and tibia, to maintain the stability of the knee joint and to limit the forward movement of the tibia when performing movements that need to change direction. If there is too much pressure on the knee, the ACL may be damaged or broken completely. Post-traumatic osteoarthritis often occurs regardless of surgical intervention following ACL injury, if left untreated, not only the knee joint will become unstable, but also aggravate the abrasion of articular cartilage and meniscus [1].

The most effective and mature treatment for ACL tears is ACL reconstruction. The reconstruction of ACL has become one of the most common operations in orthopedics and sports medicine. Although arthroscopic ACL reconstruction is very mature, the failure rate of arthroscopic reconstruction remains 6%-12% [2].

Due to the biomechanical properties of ACL reconstruction grafts, ACL reconstruction is not an immediate surgical operation. It requires a long recovery process and the entire recovery cycle is as long as two years [3]. The common reasons for the early failure of ACL reconstruction surgery are poor surgical techniques, failure of graft maturation, and wrong postoperative rehabilitation methods. Graft disruption is the disruption of the graft fiber continuity. The causes of late onset failure include re-injury after transplantation, graft disruption, etc. [4]. Therefore, follow-up in the postoperative rehabilitation process has become an important part to evaluate the situation of the patient's recovery, and clinicians will adjust the corresponding rehabilitation plan according to the results of the patient's follow-up. Postoperative follow-up mainly includes clinical physical examination and imaging investigation. Physical examination is limited by the clinician's experience and subjective judgment, and it cannot truly reflect the biomechanical properties of the graft and the postoperative recovery, but Imaging can provide objective evidence for clinicians. Scholars such as Wilson et al., Clatworthy et al. and Fahey et al. [5–7] proposed that the graft is most likely to be damaged in 4–8 months after the reconstruction, and the enlargement of the inner diameter of the tunnel occurred in 3 months after the operation. Previous studies have described imaging features linked to the stability of the knee and ACL injury [8].

In this study, the basic imaging manifestation in the early stage of postoperative patient was summarized and analyzed by reexamining MRI within one week after operation, comparing the correlation between postoperative MRI evaluation and clinical assessment, early predicting the stability of the knee joint after ACL reconstruction and providing an objective evidence for the clinic, preventing and treating postoperative complications as soon as possible, and increasing the probability of success after surgery and improving patient quality of life.

## Materials and Methods

### Research object

**Fig. 1:**
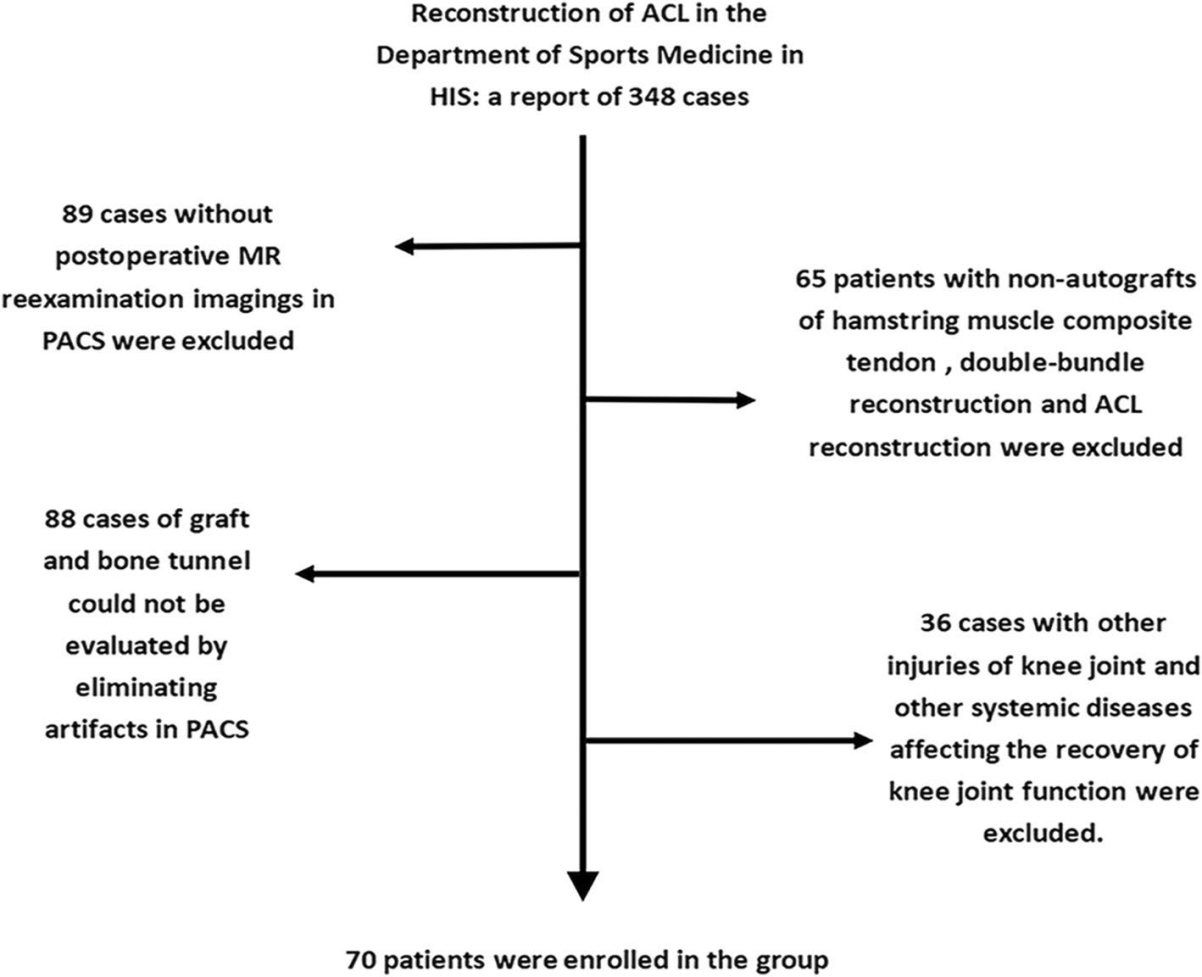
Inclusion process of enrolled patients

A total of 348 patients were collected who underwent ACL reconstruction operation in the Sports Medicine Department of the First Affiliated Hospital of Kunming Medical University from January 2016 to December 2019. Among them, 223 patients were selected who underwent MRI within one week after surgery. The inclusion and exclusion criteria of patients are shown in (Fig. 1). After exclusion of unsuitable patients, a total of 70 patients were included in this study.

#### Patient inclusion criteria

1. After ACL injury and arthroscopic ACL reconstruction, the patient's sex and age were not limited;
2. ACL reconstruction of unilateral knee joint only;
3. The reconstruction autograft is a composite tendon of semitendinosus and gracilis;
4. Clinical physical examination after ACL reconstruction: assessment of knee joint function and stability.

#### Patient exclusion criteria

1. Patients with other important organ injuries such as brain, chest and abdomen before operation;
2. Combined with other injuries of the knee joint, such as posterior cruciate ligament injury, medial and lateral ligament injury, meniscus injury and so on, which will affect the stability of the knee joint.
3. Combined with other systemic diseases, such as rheumatoid arthritis, gouty arthritis, etc., which will affect functional recovery.
4. Re-rupture of the graft caused by secondary injury after surgery.
5. The patient underwent ACL revision reconstruction.
6. Patients with complications such as infection after surgery.
7. Those patients who cannot evaluate bone tunnels and intra-articular grafts due to artifacts.

#### Clinical assessment criteria and grouping of knee joint function and stability

All selected patients underwent clinical knee joint stability examination before MR:

Lachman test [9]: The patient lay flat on the examination bed, keep their muscles relaxed, make their injured knee flexed to 20 ~ 30 degrees and feet flat; Then the examiner uses one hand to stabilize the distal femur while using the other hand to grasp the proximal tibia; Next, push back and forth in the opposite direction; Compared with the healthy side, if there is more forward movement than the healthy side, it is regarded as positive. If the Lachman test is positive, it is considered that there is instability in the knee joint. Take the patient's clinical assessment as the standard, patients were divided into stable knee joint group and unstable knee joint group according to postoperative mobility restriction or pathologic laxity of the knee joint.

### MRI Image acquisition

#### Scanning equipment and experimental process

Three different MRI machines are usedin this study: Philips superconducting magnetic resonance (Achieva, Philips, Best, Netherlands 3.0 T), GE (Discovery MR 750 3.0 T) and GE 1.5 T Magnetic Resonance Scanner (SignaHDxt), Knee joint coil, supine position, foot side first. The scanning sequences included: Axial T2WI FS, Sagittal PDWI FS, Sagittal T1WI and Coronal PDWI FS. Specific scanning parameters for each machine are as follows: (1) Philips 3.0 T with 8-channel knee coils: ①Axial T2WI FS: TR = 2425 ms, TE = 65 ms, FOV = 150 × 162mm^2^, Matrix = 316 pixels × 209 pixels, Phase encoding direction: R >  > L, NEX = 2, Acquisition time = 2min11s. ②Sagittal PDWI FS: TR = 4203 ms, TE = 30 ms, FOV = 180 × 190mm^2^, Matrix = 300 pixels × 250 pixels, Phase encoding direction: F >  > H, NEX = 1, Acquisition time = 2min56s. ③Sagittal T1WI: TR = 633 ms, TE = 20 ms, FOV = 190 × 180mm^2^, Matrix = 456 pixels × 355 pixels, Phase encoding direction: F >  > H, NEX = 1, Acquisition time = 1min48s. ④Coronal PDWI FS: TR = 3329 ms, TE = 30 ms, FOV = 190 × 171mm^2^, Matrix = 316 pixels × 209 pixels, Phase encoding direction: R >  > L, NEX = 2, Acquisition time = 2min19s. (2) GE 3.0 T with 16-channel knee coils: ①Axial T2WI FS: TR = 2710 ms, TE = 48 ms, FOV = 160 × 160mm^2^, Matrix = 320 pixels × 224 pixels, Phase encoding direction: R >  > L, NEX = 2, Acquisition time = 1min20s. ②Sagittal PDWI FS: TR = 2693 ms, TE = 35 ms, FOV = 160 × 160mm^2^, Matrix = 320 pixels × 224 pixels, Phase encoding direction: F >  > H, NEX = 2, Acquisition time = 2min25s. ③Sagittal T1WI: TR = 718 ms, TE = 13 ms, FOV = 160 × 160mm^2^, Matrix = 320 pixels × 224 pixels, Phase encoding direction: F >  > H, NEX = 1, Acquisition time = 1min15s. ④Coronal PDWI FS: TR = 2354 ms, TE = 35 ms, FOV = 160 × 160mm^2^, Matrix = 320 pixels × 224 pixels, Phase encoding direction: R >  > L, NEX = 2, Acquisition time = 2min15s. (3) GE 1.5 T with HD Trknee PA: ①Axial T2WI FS: TR = 2540 ms, TE = 60 ms, FOV = 170 × 170mm^2^, Matrix = 320 pixels × 224 pixels, Phase encoding direction: S >  > I, NEX = 2, Acquisition time = 2min16s. ②Sagittal PDWI FS: TR = 2900 ms, TE = 30 ms, FOV = 170 × 170mm^2^, Matrix = 320 pixels × 224 pixels, Phase encoding direction: A >  > P, NEX = 2, Acquisition time = 2min30s. ③Sagittal T1WI: TR = 500 ms, TE = 10 ms, FOV = 170 × 170mm^2^, Matrix = 320 pixels × 224 pixels, Phase encoding direction: A >  > P, NEX = 2, Acquisition time = 1min42s. ④Coronal PDWI FS: TR = 2540 ms, TE = 30 ms, FOV = 170 × 170mm^2^, Matrix = 320 pixels × 224 pixels, Phase encoding direction: A >  > P, NEX = 2, Acquisition time = 1min37s. The slice thickness and gap of different machines were consistent. The slice thickness was 4 mm and the slice spacing was 0.4 mm.

**Fig. 2:**
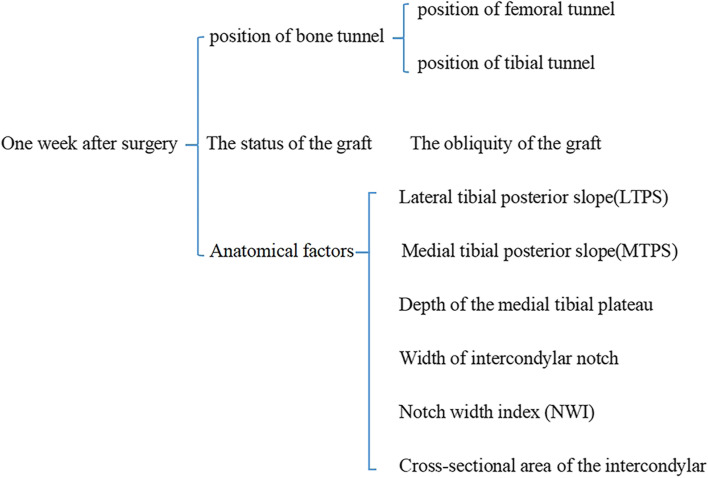
The contents of the MR examinations records of the enrolled patients

Patients enrolled in this study were examined by MRI of the knee joint within one week after operation. The experimental process is shown in (Fig. 2): The position of tibial tunnel and femoral tunnel were recorded within one week after operation; Coronal and sagittal obliquity of grafts; Anatomical factors, including: the lateral tibial posterior slope (LTPS), the medial tibial posterior slope (MTPS), the depth of the medial tibial plateau, the width of intercondylar notch, the cross-sectional area of the intercondylar notch, the notch width index (NWI).The above data were evaluated and recorded by two physicians in the musculoskeletal imaging diagnosis group.

#### MR image analysis

The evaluation criteria of imaging evaluation indicators related to surgery are as follows:

##### Position of bone tunnel

The Sagittal PDWI FS series images were used to evaluate the position of the femoral and tibial bone tunnels one week after the operation.

**Position of femoral tunnel**: Firstly, the lateral femoral condyle was selected to display the first slice of the graft tunnel; Secondly, the height and width of the lateral femoral condyle at this slice were measured; Then, the distance between the center of the tunnel and the lower and posterior edges of the lateral femoral condyle were measured, and compared it with the height and width of the lateral femoral condyle as the height ratio and the width ratio of the intra-femoral graft tunnel; Finally, the position of the intra-femoral tunnel were defined in the anterior and posterior, superior and inferior position of the lateral femoral condyle [10].

**Anchoring point opening of femoral tunnel**: On the sagittal plane of the knee joint, it was located at the intersection of the lateral wall of the intercondylar notch and the posterior femoral cortex [11]. On the coronal plane of the knee joint, the position of the opening of femoral tunnel was measured by the clock face method centered on the intercondylar notch. The femoral tunnel of the right knee joint approximately opens at 10–11 o'clock position; the femoral tunnel of the left knee joint approximately opens at 1–2 o'clock position [12].

**Position of tibial tunnel entrance**: On the sagittal plane, the positional relationship between the Blumensaat's line and the front border of the inner opening of the tibial tunnel was measured. The front border of the inner opening of the tibial tunnel should fall behind the tangential line of the Blumenstaat's line [13].

The distance from the inner opening center of the tibial tunnel to the anterior edge of the tibial plateau accounts for the percentage of the anteroposterior diameter of the tibial plateau. The opening center of the tibial tunnel should ideally be located around the 42% mark of the entire distance of the anteroposterior diameter of the tibial plateau on the sagittal plane [14].

**Position of tibial tunnel exit**: On the sagittal plane of the external opening of the tibial tunnel was located. Then, the distance from the upper edge of the external opening of the tibial tunnel to the cortical bone of the anterior and superior edge of the tibial plateau was measured [10].

##### The status of the graft

The Sagittal PDWI FS and Coronal PDWI FS series images were used to evaluate the status of the graft.

The obliquity of the graft:

The coronal obliquity of the graft: The angle between a line drawn along the long axis of the intra-articular graft and the plane of the tibial articular surface at the inner opening of the tibial tunnel was measured on the coronal image. The obliquity of the graft in the coronal plane should be less than 75° [15].

The sagittal obliquity of the graft: The angle between the vertical line of the tibia long axis and the intra-articular long axis of the graft on the sagittal image was measured. The sagittal obliquity of the graft after ACL reconstruction should be between 50–60°, and not exceed 60° [16].

##### Anatomical factors

The Sagittal PDWI FS and Coronal PDWI FS series images were used to evaluate the anatomical factors.

Measurement of the medial and lateral slope of tibial plateau [17, 18]: The largest slice of the tibial plateau was selected on the axial image of the knee joint and the plane with the largest anteroposterior diameter of the medial and lateral condyle was selected as the measurement plane of the medial and lateral slope of tibial plateau. The lateral tibial posterior slope (LTPS) and medial tibial posterior slope (MTPS) were measured on the corresponding sagittal images and the long axis of the tibia on the central sagittal image was determined.

The lateral tibial posterior slope (LTPS) was at the plane of the lateral tibial plateau and the angle between the vertical line of the long axis of the tibia and the cortical bone line of the posterior edge of the lateral tibial plateau was measured.

The medial tibial posterior slope (MTPS) was at the plane of the medial tibial plateau and the angle between the vertical line of the long axis of the tibia and the bone cortical line of the posterior edge of the medial tibial plateau was measured.

Measurement of the depth of the medial tibial plateau: the line connecting the cortical bone of the anterior and posterior edge of the tibial plateau was made at the maximum slice of the medial tibial plateau, and the vertical distance from the most concave point of the proximal tibial plateau to the line was Medial Tibial Depth [19].

Measurement of the width of intercondylar notch [20]: measured on the coronal image corresponding to the midpoint slice of the Blumensaat's line on the sagittal image, and crossed the popliteal groove to make the parallel line connecting the medial and lateral condyle cortex of the distal femur. The width of the parallel line was occupied by the intercondylar notch, so it's the width of the intercondylar notch [21]. The width of the intercondylar notch can be expressed by the notch width index (NWI) [22], that is, the ratio of the width of the intercondylar notch to the width of line connecting the medial and lateral femoral condyles at the level of the popliteal groove.

The cross-sectional area of the intercondylar notch: the vertical line from the top of the intercondylar notch to the line of the cortical bone of the medial and lateral condyle is the height of the intercondylar notch. The cross-sectional area of the intercondylar notch was measured by multiplying width and height.

### Statistical methods

Statistical analysis was performed using SPSS 22.0 statistical software. Independent sample t-test was used to detect the difference between the stable group and the unstable group, which includes: age, postoperative days, percentage of tibial tunnel entrance, position of tibial tunnel exit, position of femoral tunnel (the height ratio and width ratio), lateral tibial posterior slope (LTPS), medial tibial posterior slope (MTPS), Medial tibial depth, width of intercondylar notch, Notch width index (NWI), cross-sectional area of the intercondylar notch, coronal obliquity of the graft, The sagittal obliquity of the graft; The differences of the position of the entrance of the tibial and femoral tunnel between the two groups were compared by χ^2^ test;Kappa consistency test to compare the consistency between MR findings and clinical presentation, > 0.75 indicates good consistency, 0.40 ~ 0.75 indicates medium consistency, < 0.40 indicates poor consistency. The sensitivity, specificity, accuracy, positive predictive value and negative predictive value of the above MR findings in the diagnosis of postoperative stability of knee joint were calculated, *P* < 0.05 was considered that the difference is statistically significant. The independent predictors are screened by logistic regression analysis, and the prediction equation is constructed at the same time, *P* < 0.05 was considered that the difference is statistically significant. ROC curve was used to compare the prediction efficiency of independent prediction factors and prediction equations. We used t test, rank sum test and chi-square test to select the variables with statistically significant differences, then significant variables were passed to a secondary multivariate logistic regression model.

## Results

### Clinical evaluation and grouping

A total of 70 patients were enrolled in this study. Among them, there were 48 males and 22 females, aged from 14 to 64 years old, with a mean age of 32.5 ± 10.69 years old. Clinical follow-up within two years,13 cases had positive Lachman test after reconstruction of ACL denoting unstable knee joint on clinical assessment. Thus, these patients were included in postoperative unstable knee group; the remaining 57 cases were included in the postoperative stable knee group. The analysis of the basic information between the stable and unstable knee joint groups is shown in (Table 1). After statistical analysis, patient's age, sex, surgical site and the time of postoperative reexamination imaging, there were no statistical and mathematical differences between the stable knee joint group and the unstable group after ACL reconstruction (*P* > 0.05).

**Table 1:** Analysis of basic conditions between stable and unstable knee groups after ACL reconstruction

Baseline	Stable group (*n* = 57)	Unstable group (*n* = 13)	*P*
Age (mean ± SD)	32.72 ± 10.208	33.46 ± 13.068	0.823
Sex			0.349
Male	41 (72%)	7 (54%)	
Female	16 (28%)	6 (46%)	
Body parts			0.425
Left knee joint	33 (58%)	9 (69%)	
Right knee joint	24 (42%)	4 (31%)	
The interval of the first postoperative reexamination, days	4 (3,4)	4 (3,4,5)	0.273
P50 (P25, P75)			

### MR image analysis: comparison of the first reexamination findings within one week after ACL reconstruction

The comparison of MRI findings within one week after ACL reconstruction is shown in (Table 2). According to statistical analysis, there were significant differences in the abnormal position of tibial tunnel entrance, percentage of the position of tibial tunnel entrance, position of tibial tunnel exit, lateral tibial posterior slope (LTPS) and the width of the intercondylar notch between stable knee joint group and unstable knee joint group after ACL reconstruction (*P* < 0.05).

**Table 2:** Statistical analysis of magnetic resonance imaging findings in stable group and unstable group after operation (within one week)

MRI findings	Stable group (*n* = 57)	Unstable group (*n* = 13)	Statistic quantity	*P* value
Abnormal position of tibial tunnel entrance, n (%)	4(7.02%)	6(46.15%)	χ^2^ = 2.169	0.002
Inner opening of the tibial tunnel (%), mean ± SD	30.438 ± 6.620	26.308 ± 6.143	t = -2.056	0.044
Position of tibial tunnel exit (mm), mean ± SD	20.091 ± 5.033	13.7638 ± 6.009	t = -3.954	0.001
Abnormal position of femoral tunnel entrance, n (%)	27(47.37%)	10(76.92%)	χ^2^ = 3.711	0.054
Height ratio of the position of femoral tunnel, mean ± SD	0.747 ± 0.122	0.698 ± 0.115	t = -1.328	0.189
Width ratio of the position of femoral tunnel, mean ± SD	0.265 ± 0.065	0.314 ± 0.128	t = 1.339	0.203
Lateral tibial posterior slope (LTPS) (°) mean ± SD	6.667 ± 3.061	10.077 ± 3.201	t = 3.595	0.001
Medial tibial posterior slope (MTPS) (°), mean ± SD	7.526 ± 2.707	9.231 ± 3.244	t = 1.974	0.052
Depth of the medial tibial plateau (mm), mean ± SD	2.421 ± 0.691	2.716 ± 0.947	t = 1.291	0.201
Width of intercondylar notch (mm), mean ± SD	14.937 ± 2.589	13.353 ± 2.392	t = -2.016	0.048
Intercondylar Notch width index (NWI)	0.214 ± 0.032	0.197 ± 0.032	t = -1.725	0.089
Cross-sectional area of the intercondylar notch (mm^2^), mean ± SD	307.070 ± 63.924	274.462 ± 47.330	t = -1.730	0.088
Coronal obliquity of the graft (°), mean ± SD	61.421 ± 6.375	59.769 ± 7.876	t = -0.806	0.423
Sagittal obliquity of the graft (°), mean ± SD	52.439 ± 7.246	48.231 ± 10.655	t = -1.721	0.090

### Comparison of the consistency between MR findings and clinical evaluation

Positive MRI findings after ACL reconstruction to evaluate the stability of knee joint after operation is shown in (Table 3). According to statistical analysis, the positive MRI findings that the position of tibial tunnel entrance, percentage of the position of tibial tunnel entrance, position of tibial tunnel exit, lateral tibial posterior slope (LTPS), width of intercondylar notch were used to evaluate the function of knee joint. There were significant differences between stable group and unstable group (*P* < 0.05).

**Table 3:** Positive MRI findings after ACL reconstruction to evaluate the stability of knee joint after operation

Statistical evaluation							
MRI findings	Sensitivity (%)	Specificity (%)	consistency rate (%)	Positive predictive value	Negative predictive value	Kappa	*P*
Position of tibial tunnel entrance	60.00 (6/10)	88.33 (53/60)	84.29 (59/70)	46.15 (6/13)	92.98 (53/57)	0.430	0.000
Percentage of the position of tibial tunnel entrance	30.00 (6/20)	86.00 (43/50)	70.00 (49/70)	46.15 (6/13)	75.44 (43/57)	0.179	0.020
Position of tibial tunnel exit	27.03 (10/37)	90.91 (30/33)	57.14 (40/70)	76.92 (10/13)	52.63 (30/57)	0.173	0.044
Lateral tibial posterior slope (LTPS)	58.33 (7/12)	89.66 (52/58)	84.29 (59/70)	53.85 (7/13)	91.23 (52/57)	0.465	0.000
Width of intercondylar notch	33.33 (9/27)	90.70 (39/43)	68.57 (48/70)	69.23 (9/13)	68.42 (39/57)	0.266	0.012

The positive MRI findings of the position of tibial tunnel entrance and lateral tibial posterior slope (LTPS) are generally consistent with the clinical physical examination, indicating that the two methods cannot replace each other, and the two should be evaluated comprehensively.

### Comparison of operative factors and self-anatomical factors in postoperative stability of knee joint

After the multi-factor binary logistical regression analysis, the position of tibial tunnel exits and the lateral tibial posterior slope (LTPS) and the sagittal obliquity of the graft were independent predictors among surgical factors and self-anatomical factors (*P* < 0.05), as shown in (Table 4). The OR value of the lateral tibial posterior slope (LTPS) is more than 1, indicating that it is a risk factor for postoperative stability of the knee joint. The OR values of the position of tibial tunnel exit and the sagittal obliquity of the graft were less than 1, indicating that both are the protective factors for the postoperative stability of the knee joint.

**Table 4:** Multi-factor binary logistical regression analysis of surgical factors and self-factors in postoperative Stability of knee Joint

	*P*	OR	95% confidence interval	Constant (B)
Position of tibial tunnel exit	0.005	0.794	0.677–0.931	-0.231
Lateral tibial posterior slope (LTPS)	0.003	1.663	1.192–2.319	0.509
Abnormal sagittal obliquity of the graft	0.045	0.122	0.016–0.953	-2.105

OR > 1 risk factor, OR < 1 protective factor. According to statistical analysis, lateral tibial posterior slope (LTPS) is a risk factor for postoperative stability of knee joint. The position of tibial tunnel exit and the sagittal obliquity of the graft were protective factors for the stability after knee joint operation (*P* < 0.05).

Based on multi-factor logistical regression, the prediction equation of the postoperative stability of knee joint was established:

$$\mathrm{Logit}(\mathrm P)\:=\:-1.067-0.231\ast\mathrm{position}\;\mathrm{of}\;\mathrm{tibial}\;\mathrm{tunnel}\;\mathrm{exit}+0.509\ast\mathrm{lateral}\;\mathrm{tibial}\;\mathrm{posterior}\;\mathrm{slope}\;(\mathrm{LTPS})-2.105\ast\mathrm{sagittal}\;\mathrm{obliquity}\;\mathrm{of}\;\mathrm{the}\;\mathrm{graft}.$$

### ROC curve compares the prediction efficiency of the prediction factor and the prediction equation

**Fig. 3:**
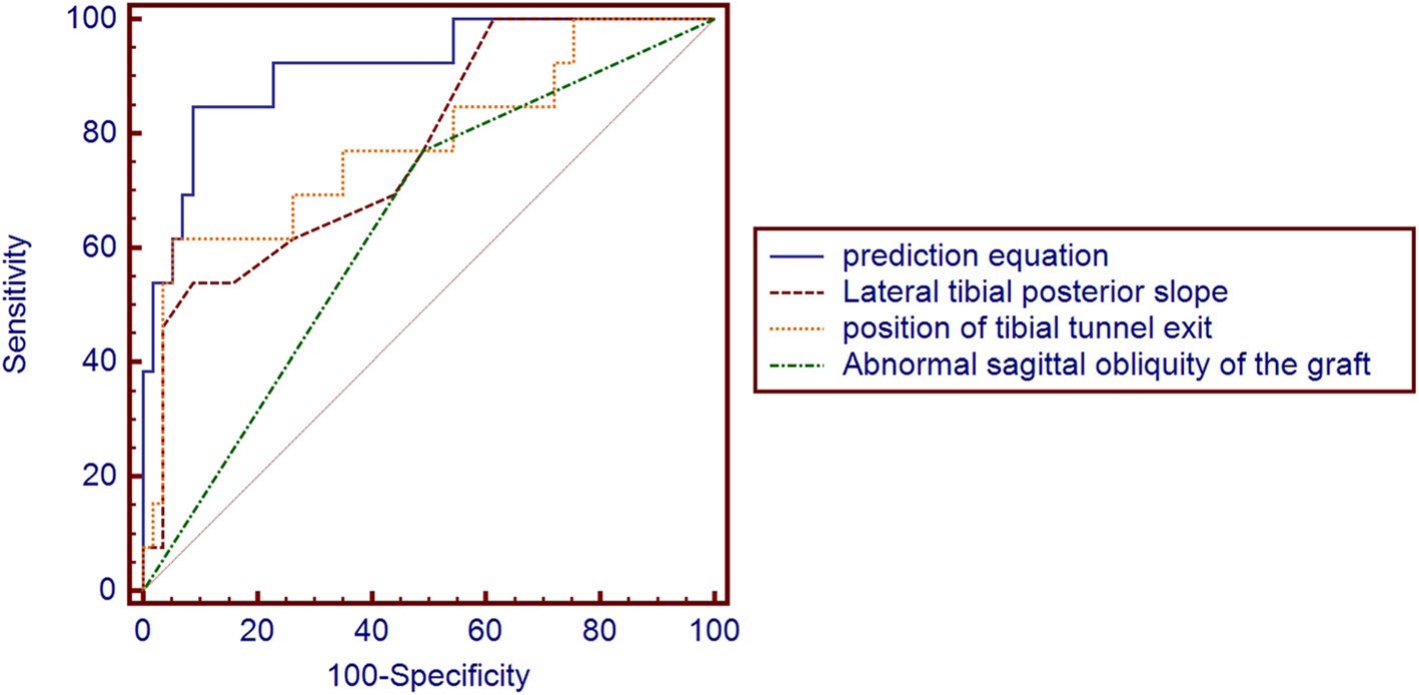
ROC curve of prediction equation, surgical factors and self-anatomical factors

The ROC curve was constructed by the prediction equation, lateral tibial posterior slope (LTPS), position of tibial tunnel exit and the sagittal obliquity of the graft, as shown in (Fig. 3) and (Table 5).

**Table 5:** ROC curve analysis of prediction equation, lateral tibial posterior slope (LTPS), position of tibial tunnel exit and sagittal obliquity of the graft

	AUC	Sensitivity	Specificity	Positive predictive value	Negative predictive value	Kappa	*P*	95% confidence interval
Prediction equation	0.915	0.846	0.912	0.688	0.963	0.696	< 0.001	0.825–1.000
Position of tibial tunnel exit	0.779	0.615	0.947	0.727	0.915	0.598	< 0.001	0.623–0.935
Lateral tibial posterior slope (LTPS)	0.775	0.538	0.912	0.583	0.897	0.465	0.001	0.637–0.913
Abnormal sagittal obliquity of the graft	0.639	0.769	0.509	0.263	0.935	0.16	0.069	0.479–0.799

The prediction equation predicted that the AUC of knee instability was 0.915, the sensitivity was 84.6%, and the specificity was 91.2%. The AUC of the position of tibial tunnel exit, lateral tibial posterior slope (LTPS) and abnormal sagittal obliquity of the graft to predict knee instability were all less than 0.80.

## Discussion

This study found that the early positive findings of knee instability after anterior cruciate ligament reconstruction using magnetic resonance includes three factors: the position of tibial tunnel exit, the lateral tibial posterior slope (LTPS) and the sagittal obliquity of the graft. The study used a predictive model composed of the above factors to predict knee instability after ACL reconstruction, and the AUC was 0.915.

The incidence rate of ACL injury is related to occupational and biomechanical characteristics at the time of injury. Shelbourne pointed out that [23] the incidence rate of ACL injury in the general population is about 38/100000, and that of professional athletes is about 60–70/100000, with more women than men. The failure of ACL reconstruction is marked by pathological relaxation of graft or limitation of joint movement [4]. The incidence rate of primary knee instability after ACL reconstruction was 3%—10% [24]. The graft is most prone for injury 4–8 months after reconstruction, and the vast majority of early surgical failures occur within half a year after operation, so it is particularly important to predict knee joint stability after ACL reconstruction by early postoperative MRI findings.

The surgical factors that affect the stability of the knee joint after ACL reconstruction and the patient's own anatomical factors were summarized in this study. The positive findings of MR were used to predict the stability of the knee joint within one week after the operation. According to statistical analysis, there were significant differences in the abnormal position of tibial tunnel entrance, percentage of the position of tibial tunnel entrance, position of tibial tunnel exit, lateral tibial posterior slope (LTPS), width of intercondylar notch between stable knee joint group and unstable knee joint group after ACL reconstruction (*P* < 0.05). After the multi-factor binary logistical regression analysis, the position of tibial tunnel exit and the lateral tibial posterior slope (LTPS) and the sagittal obliquity of the graft were independent predictors among surgical factors and self-anatomical factors (*P* < 0.05). Based on multi-factor logistical regression, the prediction equation of postoperative stability of knee joint was established. Logit(P) = -1.067–0.231*position of tibial tunnel exit + 0.509*lateral tibial posterior slope (LTPS)-2.105*sagittal obliquity of the graft. The prediction equation predicted that the AUC of knee instability was 0.915, the sensitivity was 84.6%, and the specificity was 91.2%. The AUC of the position of tibial tunnel exit, the lateral tibial posterior slope (LTPS) and the abnormal sagittal obliquity of the graft to predict knee instability were all less than 0.80. Thus, it can be said that the prediction equation of knee joint postoperative stability is more effective in predicting knee joint instability.

For ACL reconstruction, the entrance of the femoral tunnel should be should be located at the intersection of the lateral wall of the intercondylar notch and the posterior femoral cortex on the sagittal position, and the position of the inner femoral tunnel should be at the back and upper part of the lateral condyle of the femur, that is, the height ratio of the graft in the first slice of the lateral condyle of the femur should be larger and the width ratio. Although statistics showed that there was little correlation between the height ratio and width ratio of the femoral tunnel and the stability. This data is directly related to the inner opening of the tibial tunnel and the obliquity of the graft, and these two factors are related to stability. The front border of the opening of the tibial tunnel should fall behind the tangential line of the Blumenstaat's line. This position is approximately 42% of the anteroposterior diameter of the tibial plateau. The front of the tibial entrance tunnel is the main factor causing the graft impingement. If the tibial tunnel is too forward (partly or entirely in front of the Blumenstaat's line), the graft will hit the top of the intercondylar notch. The tibial tunnel is not near the intercondylar eminence and the graft will hit the lateral wall of the intercondylar notch [14, 25, 26]. The tibial tunnel exit should not be too close to the articular surface of the anterior upper edge of the tibia. If the tibial tunnel exit being too close to the articular surface can cause the tunnel and graft to lean backward, increasing the risk of instability, especially on the sagittal plane, which makes the graft lose its biomechanical properties, and it is easy to cause fractures of the tibial plateau. Because of the tunnel through the area, the cortical bone becomes relatively thinner. If the distance from the tibial tunnel exit to the articular surface of the anterior upper edge of the tibia is too close, the bones are vulnerable to secondary fractures. In this study, the probability of abnormal position of bone tunnel in knee instability group was higher, the sensitivity and specificity of knee instability caused by abnormal position of bone tunnel were higher, and the evaluation of clinical consistency was moderate. It can be concluded that the position of bone tunnel is one of the indexes to judge the stability of knee joint.

Among the abnormal position of bone tunnel, the incidence rate of abnormal position of tibial tunnel was the highest. The anterior tibial tunnel accounted for more than half of the abnormal position of the tibial tunnel. In this study, the average percentage of the inner opening of the tibial tunnel at the anteroposterior diameter of the tibial plateau in the knee instability group was about 26%, approximately at the front 1/4 of the anteroposterior diameter of the tibial plateau, while the stable group was 30%. It can be seen that the position of the inner opening of the tibial tunnel in the unstable group was significantly anterior than in the stable group. In addition, the instability accounted for 18.57% in this study, which was significantly higher than that in the literature. The anterior tibial tunnel should be an important reason.

The correct position of the femoral and tibial tunnel is very important for the stability of the graft and good clinical results, so each patient underwent ACL reconstruction should be evaluated and documented.

Tomczak, Sanders et al. [11, 13] pointed out that the anterior position of the tibial tunnel is the most likely to cause the intercondylar notch impingement. In the case of intercondylar notch impingement, MRI showed an increase in signal intensity at the injured site, mostly in the first two thirds of the graft, which was different from the increased signal intensity of vascularization and synovialization of the graft during the postoperative recovery period. Because the position of increased signal caused by the impingement is more limited, if the anatomical risk factors are not removed, the high signal of the impingement will always exist, which will cause a tear in the graft.

Papakonstantino believes that [12] when the femoral tunnel is too far forward, the length and tension of the graft will increase when the knee is bent, which will increase the risk of injury. In this study, the intercondylar notch impingement occurred in the anterior graft of the tibial tunnel, and the impingement site was near the midpoint of the graft, and the localized signal increased during the follow-up. In the knee joint stability group, the reason why there was no impingement due to the forward movement of the femoral tunnel and tibial tunnel may be related to the anatomical morphology of the intercondylar notch, and the impingement is more likely to occur in the narrow intercondylar notch.

The position of the inner opening of the tibial and femoral tunnel determines the obliquity of the graft. Mall and Saupe [15, 16] proposed that the posterior obliquity of the graft should be between 50°- 60°on the sagittal plane, and should not exceed 60°on the sagittal plane, and the coronal obliquity of the graft should be less than 75°. If the limit is exceeded, the graft will relax. In this study, the average of the sagittal obliquity of the graft in the unstable group was 48°only, and the minimum value was 24°, which was equivalent to lying horizontally in the articular cavity, and made the reconstructed ligament lose its biomechanical function.

The impingement of the graft will cause a variety of complications, including graft tear, graft fibrosis, graft myxoid degeneration. The most common impingement site is the impingement between the Blumenstaat's line and the middle and lower part of the graft, which is characterized by increased local signal and local edema.

Fibrosis of the graft is the excessive proliferation of fibrous tissue in the joint cavity after surgery [12, 13, 27, 28]. The fibrotic area limits the movement of the knee joint, which is the main reason for the limitation of knee joint extension. Two types of fibrosis can be observed on MRI; diffuse fibrosis and localized fibrosis. Localized fibrosis is the most common complication of ACL reconstruction. Low signal nodules in the distal anterior part of the graft can be seen on magnetic resonance images, which is called "cyclops sign". Diffuse fibrosis is characterized by synovial thickening around the graft, because of the infiltration of inflammatory cells, which can extend to the articular capsule, resulting in the thickening of the articular capsule. Fibrosis is caused by the injury of the graft, which can cause mechanical disturbance of the end of the graft. The areas with low signal of T1WI and low signal of T2WI were showed on MR.

In the process of graft maturation, the continuity of the graft should be kept intact in order to maintain the stability of the knee joint. However, if the knee joint is injured again in the process of rehabilitation, the increased signal of the injury site overlaps with the high signal in the process of ligamentization, which will increase the difficulty for diagnosis. Therefore, we need to pay attention to the continuity of the graft. If there is a discontinuity of the tendon fiber bundle, it indicates the injury or tear of the reconstructed ligament. Horton [29] suggested that the continuous coronal image of ligament is more accurate.

**Fig. 4:**
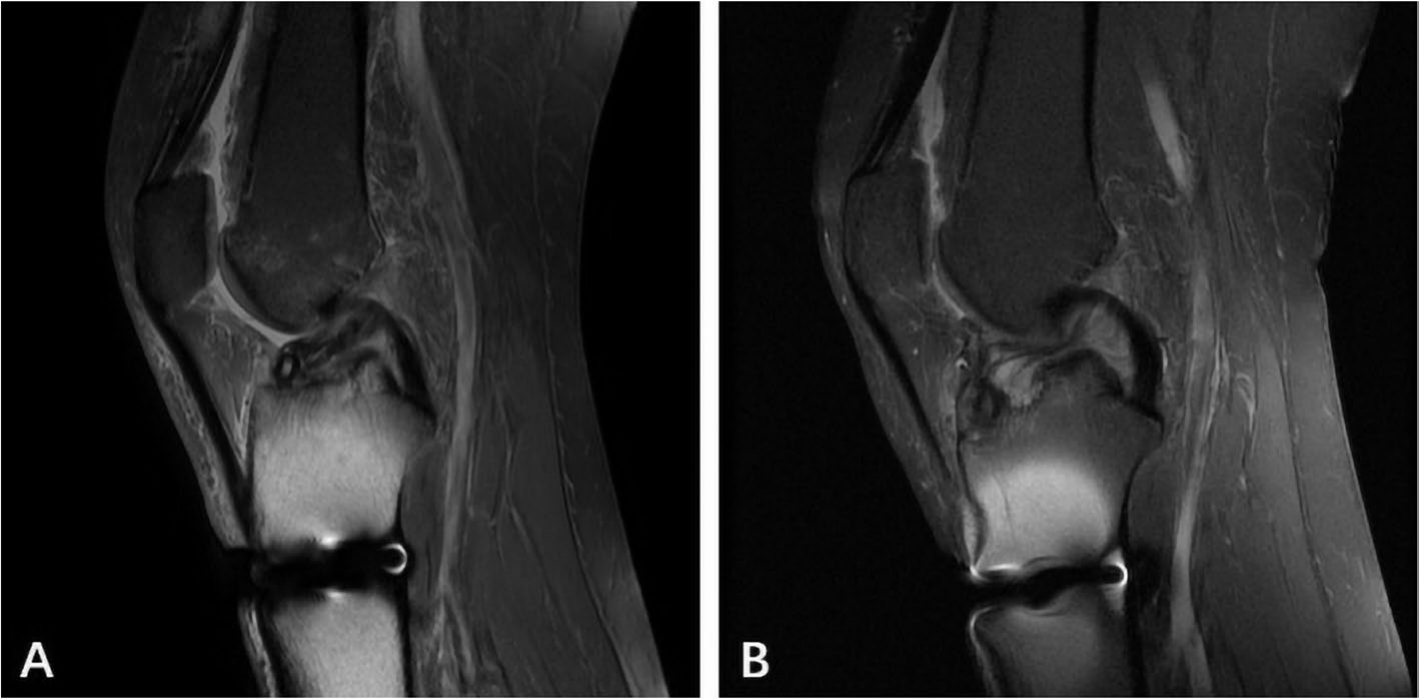
49-year-old female patients with ACL reconstruction: **a** one week after operation, MRI showed that the inner opening of tibial tunnel was anterior and the lateral tibial posterior slope (LTPS) was 14°; **b** half a year after operation, the continuity of ACL reconstruction was interrupted and fluid was accumulated at the internal opening of tibial tunnel

The patient's own anatomical factors also have a certain influence on the stability of postoperative knee joint function. Some scholars have found that [30–32]: Narrow intercondylar notch, increased tibial posterior slope (TPS) and deeper medial tibial plateau all affect the biomechanical properties of knee joint. These three factors are already the risk factors of ACL injury in normal human knee joint injury. It has been confirmed that the tibial posterior slope (TPS) increases, especially the lateral tibial posterior slope (LTPS). When it is more than 10°, the risk of graft injury after ACL reconstruction is significantly increased. As shown in (Fig. 4), this study also confirmed that there was a significant difference in the lateral tibial posterior slope (LTPS) between the stable and unstable groups of the knee joint, with no difference between male and female. If the lateral tibial posterior slope (LTPS) increases, and the risk of knee instability increases. There was no significant difference in anatomical risk factors between males and females in this group.

**Fig. 5:**
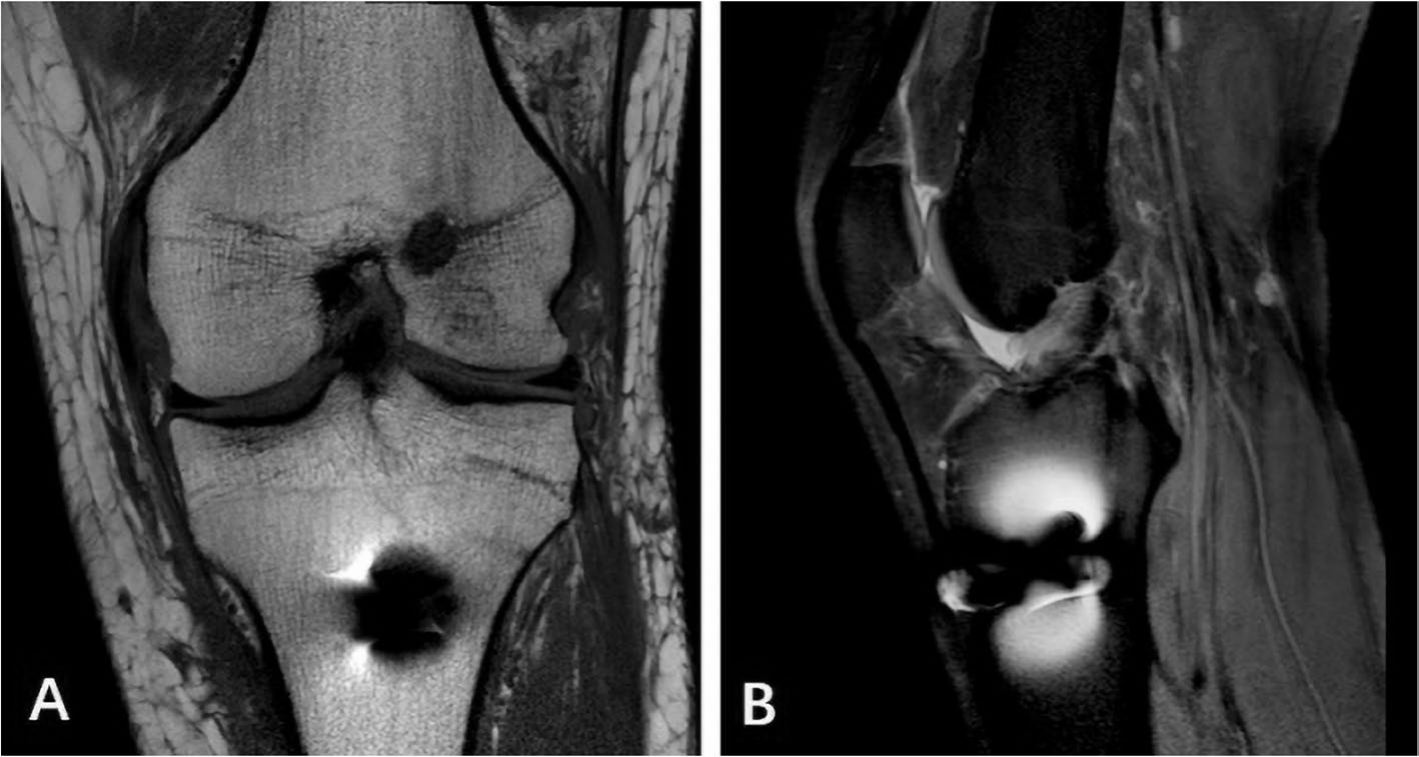
Postoperative follow-up of 26-year-old male patients with ACL reconstruction: **a** Reexamination of magnetic resonance imaging one week after operation, the intercondylar notch width index (NWI) was 0.19; **b** half a year after operation, MRI showed that reconstruction of ACL impingement and peripheral synovial hyperplasia

The risk of graft impingement in narrow intercondylar notch was also significantly increased. Fujii et al. [22] have shown that when the intercondylar notch width index (NWI) is less than 0.21, the risk of graft impingement after reconstruction significantly increases as shown in (Fig. 5).

It can be seen that the preoperative evaluation of the anatomical factors of the knee joint is beneficial to the formulation of the surgical approach and provides objective evidences for the correction of the tibial posterior slope (TPS) or the plasty of the intercondylar notch. Postoperative imaging evaluation can early indicate the possibility of clinical complications.

This study has the following limitations:(1) The number of this sample was small; (2) This study was a single-center retrospective study. However, our research results are helpful to the multi-center, prospective, large sample research design in the future, so as to verify our research results. (3) Our prediction equation was obtained from the experimental data of the experimental group of this study, and it should only be applicable to the predictive analysis of patients in this experimental group. Therefore, independent new experimental data should be used to verify the prediction performance of this model.

In conclusion, we found that abnormalities of the position of the exit of the bone tunnel, lateral tibial posterior slope (LTPS) and sagittal obliquity of the graft were the early MRI positive findings of knee instability after ACL reconstruction. It is helpful for clinicians to predict the stability of knee joint after ACL reconstruction, to deal with postoperative complications as soon as possible, so as to increase the success rate of operation and improve the quality of life of patients.

## Data Availability

The datasets used and/or analysed during the current study are available from the corresponding author on reasonable request.
